# Advances in Dietary Phenolic Compounds to Improve Chemosensitivity of Anticancer Drugs

**DOI:** 10.3390/cancers14194573

**Published:** 2022-09-21

**Authors:** Abdelhakim Bouyahya, Nasreddine El Omari, Saad Bakrim, Naoufal El Hachlafi, Abdelaali Balahbib, Polrat Wilairatana, Mohammad S. Mubarak

**Affiliations:** 1 Laboratory of Human Pathologies Biology, Department of Biology, Faculty of Sciences, Mohammed V University, Rabat 10106, Morocco; 2 Laboratory of Histology, Embryology, and Cytogenetic, Faculty of Medicine and Pharmacy, Mohammed V University, Rabat 10100, Morocco; 3 Geo-Bio-Environment Engineering and Innovation Laboratory, Molecular Engineering, Biotechnologies, and Innovation Team, Polydisciplinary Faculty of Taroudant, Ibn Zohr University, Agadir 80000, Morocco; 4 Microbial Biotechnology and Bioactive Molecules Laboratory, Sciences and Technologies Faculty, Sidi Mohmed Ben Abdellah University, Fez 30050, Morocco; 5 Laboratory of Biodiversity, Ecology, and Genome, Faculty of Sciences, Mohammed V University, Rabat 10056, Morocco; 6 Department of Clinical Tropical Medicine, Faculty of Tropical Medicine, Mahidol University, Bangkok 10400, Thailand; 7 Department of Chemistry, The University of Jordan, Amman 11942, Jordan

**Keywords:** cancer, chemotherapy, drugs resistance, dietary phenolic compounds, drugs sensibilization, combination treatment

## Abstract

**Simple Summary:**

Several dietary phenolic compounds isolated from medicinal plants exert significant anticancer effects via several mechanisms. They induce apoptosis, autophagy, telomerase inhibition, and angiogenesis. Certain dietary phenolic compounds increase the effectiveness of drugs used in conventional chemotherapy. Some clinical uses of dietary phenolic compounds for treating certain cancers have shown remarkable therapeutic results, suggesting effective incorporation in anticancer treatments in combination with traditional chemotherapeutic agents.

**Abstract:**

Despite the significant advances and mechanistic understanding of tumor processes, therapeutic agents against different types of cancer still have a high rate of recurrence associated with the development of resistance by tumor cells. This chemoresistance involves several mechanisms, including the programming of glucose metabolism, mitochondrial damage, and lysosome dysfunction. However, combining several anticancer agents can decrease resistance and increase therapeutic efficacy. Furthermore, this treatment can improve the effectiveness of chemotherapy. This work focuses on the recent advances in using natural bioactive molecules derived from phenolic compounds isolated from medicinal plants to sensitize cancer cells towards chemotherapeutic agents and their application in combination with conventional anticancer drugs. Dietary phenolic compounds such as resveratrol, gallic acid, caffeic acid, rosmarinic acid, sinapic acid, and curcumin exhibit remarkable anticancer activities through sub-cellular, cellular, and molecular mechanisms. These compounds have recently revealed their capacity to increase the sensitivity of different human cancers to the used chemotherapeutic drugs. Moreover, they can increase the effectiveness and improve the therapeutic index of some used chemotherapeutic agents. The involved mechanisms are complex and stochastic, and involve different signaling pathways in cancer checkpoints, including reactive oxygen species signaling pathways in mitochondria, autophagy-related pathways, proteasome oncogene degradation, and epigenetic perturbations.

## 1. Introduction

Cancer is a significant issue for physicians in multidisciplinary health care facilities. It is a complex and multifactorial pathology in which normal cells develop mutations in their genetic structure, resulting in continued cell growth, colonization, and metastasis to other organs such as the liver, prostate, breast, lungs, brain, and colon. The transformation mechanisms range from genetic and hormonal disturbances to environmental inducers and metabolic deregulations. This divergence of risk factors gives rise to various forms of cancer and, sometimes, implies therapeutic specificity even for the same type of cancer [1,2,3]. In this regard, searching for anticancer treatments requires screening several chemical molecules with functional diversity. Among the candidate molecules studied are phenolic compounds. Chemically defined as having a phenolic structure, phenolic compounds are well recognized for their extensive pharmacological properties such as anti-inflammatory, antibiotic, antiseptic, antitumor, antiallergic, cardioprotective, etc. Phenolic compounds are derived from edible plants, particularly medicinal and aromatic plants, in many food products such as vegetables, cereals, legumes, fruits, nuts, and certain beverages. Indeed, this chemical family constitutes a group of substances frequently present in the metabolism of medicinal plants and contains several subclasses, such as acids, flavonoids, and tannins, which are the most abundant molecules [4,5,6]. Various investigations have focused on phenolic compounds as anticancer bioactive compounds. These groups of molecules exert anticancer properties by acting on the multiple checkpoints of cancerous cells and can induce apoptosis, autophagy, and cell cycle arrest with high specificity [7,8].

In addition, phenolic compounds exert other actions such as inhibiting telomeres, blocking their expression and inhibiting angiogenesis and metastases. On the other hand, phenolic compounds have recently been shown to act in combination with other bioactive compounds used in chemotherapy, sometimes with a potent synergistic mechanism. Recent investigations have also highlighted the sensitization action of cancer cells to chemotherapeutic treatments [9,10]. Indeed, dietary phenolic compounds can induce chemosensitivity of human cancers towards used drugs in chemotherapy via different molecular mechanisms, which include reducing the expression of a transcription factor regulating the expression of cytoprotective genes, the down-regulation of the phosphatidylinositol 3-kinase and Akt protein kinase B (PI3K/Ak) pathway, reducing p53 activation, enhancing the cytotoxicity of used drugs, decreasing Bcl-2 expression and mitochondrial membrane potential (ΔΨm) while inhibiting tumor growth, enhancing the cytotoxicity of used drugs, reducing Bcl-2 expression and mitochondrial membrane potential (ΔΨm) while inhibiting tumor growth, suppressing the expression of hypoxia-inducible factor (HIF-1α) and P-glycoprotein (P-gp) responsible for multidrug resistance, and increasing cellular apoptosis with down-regulation of p-Akt expression and up-regulation of phosphatase and tensin homolog (PTEN) expression [9,10]. Based on the previous discussion, this study aims to investigate and demonstrate the potential benefits of dietary sources, notably phenolic compounds, in managing and preventing cancer. Additionally, the current review aims to examine combining chemotherapeutic drugs with phenolic compounds and their sensitizing effects on cancer treatments to improve the effectiveness and diminish the harmful effects of anticancer bioactive compounds.

## 2. Dietary Phenolic Compounds Improving the Chemosensitivity of Anticancer Drugs

**Figure 1:**
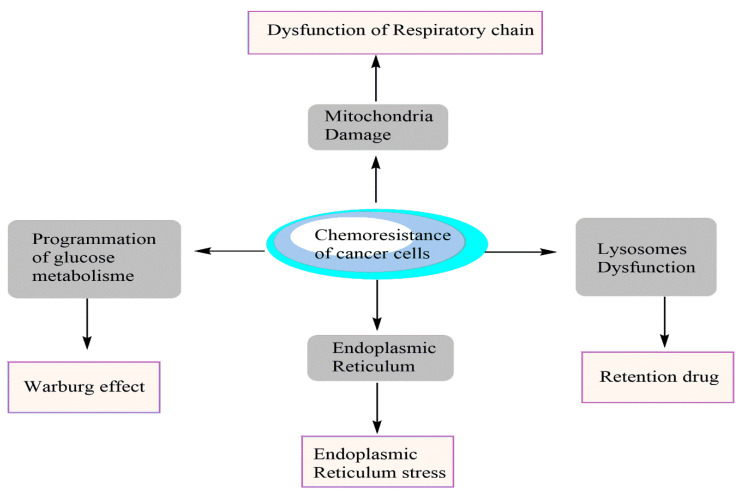
Mechanisms of chemoresistance of cancer cells against anticancer drugs.

Recent research findings showed that cancer cells could develop resistance to used drugs in chemotherapy. This resistance is related to different molecular mechanisms which give cancer cells a selective advantage in resisting drugs administered during cancer chemotherapy (Figure 1).

### 2.1. Flavonoids

Resistance to various anti-cancer treatments, whether chemotherapy or radiotherapy, remains a significant obstacle in the management of cancer patients. Therefore, the use of chemo- and radio-sensitizers of plant origin has attracted the attention of scientists to replace synthetic drugs to improve tumor sensitivity. One example of these natural compounds is flavonoids. Food products containing high levels of flavonoids include blueberries and other berries, parsley, onions, bananas, green and black tea, citrus fruits, sea buckthorn, *Ginkgo biloba*, and dark chocolate. 

**Figure 2:**
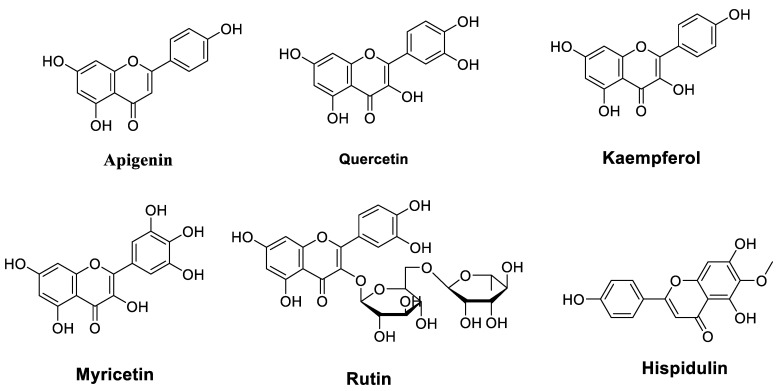
Chemical structures of flavonoids that improve the chemosensitivity of anticancer drugs.

Combinatorial treatment with flavonoids has been suggested in several studies as a potential therapeutic approach to avoid drug resistance and enhance their antitumor properties. Table 1 lists flavonoids (Figure 2) that improve the chemosensitivity of chemotherapeutic drugs in cancer.

#### 2.1.1. Flavones

Apigenin is a natural product found in numerous fruits and vegetables, but it is particularly abundant in chamomile tea, parsley, celery, propolis, and garlic oil. It was among the most investigated flavonoids in this field. In 2006, Chan, et al. [11] synthesized a series of apigenin dimers that increased the chemo-sensitivity of leukemic and breast cells, known to be multidrug-resistant (MDR), to numerous anticancer drugs, such as vinblastine (VBL), vincristine (VCR), daunomycin (DM), doxorubicin (DOX), and paclitaxel (PTX) [11]. Seven years later, the chemo-sensitive mechanism by which apigenin acts on DOX has been investigated [12]. The mechanism involves reducing the expression of a transcription factor regulating the expression of cytoprotective genes, called Nrf2, at the levels of proteins and messenger RNA by down-regulating the PI3K/Akt pathway. Compared to DOX treatment alone, the combination treatment of apigenin with DOX showed anticancer effects by inducing apoptosis, reducing cell proliferation, and inhibiting tumor growth.

**Figure 3:**
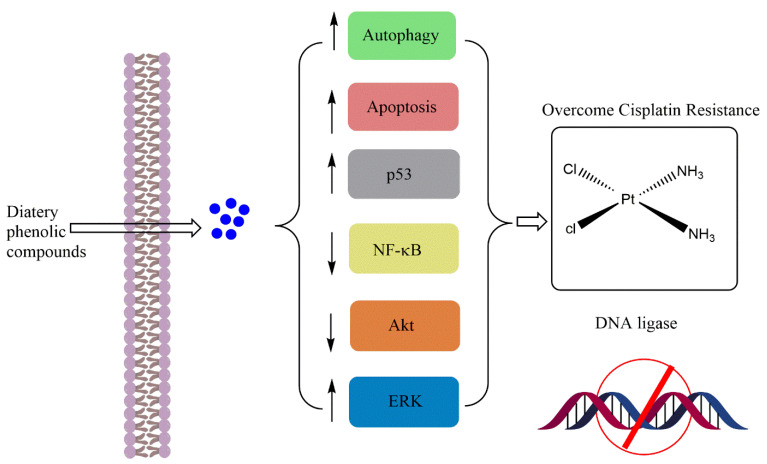
Mechanisms of chemosensitivity of apigenin towards sisplatin.

Johnson and Mejia [13] evaluated the interaction effect between this flavonoid and one of the known chemotherapeutic drugs, gemcitabine (GEM), on human pancreatic cancer cells. This interaction inhibited cell proliferation and growth by 59–73%, whereas apigenin alone potentiated the anti-proliferative effect of GEM. This effect was attributed to IKK-*β*-mediated NF-κB activation [14]. To improve the chemo-sensitivity of another chemotherapeutic agent, called cisplatin (CP), and to overcome the chemo-resistance of laryngeal carcinoma (Hep-2 cells), apigenin was chosen in an in vitro co-targeted therapy [15]. The results showed that CP-induced Hep-2 cell growth suppression was significantly enhanced in a time- and concentration-dependent manner with suppression of p-AKT and glucose transporter-1 (GLUT-1) involved in resistance to cancer treatments. Bao, et al. also tested this a year later against the same type of cancer [16]. In human renal proximal tubular epithelial cells, apigenin ameliorated CP-induced nephrotoxicity by promoting the PI3K/Akt pathway (Figure 3) and reducing p53 activation [18].

On the other hand, a promising combined effect was recorded with apigenin and 5-fluorouracil (5-FU), a chemotherapeutic drug belonging to the class of antimetabolite drugs [17,19]. In this context, apigenin significantly improved the treatment of hepatocellular carcinoma (HCC) by enhancing the cytotoxicity of 5-FU [17]. The combination of these two elements decreased Bcl-2 expression and mitochondrial membrane potential (ΔΨm) while inhibiting tumor growth of HCC xenografts. This was in agreement with the results of Gaballah and collaborators [19], who also observed a reduction in tumor size and Mcl-1 expression, with an increase in Beclin-1 levels and caspase-3 and -9 activities. Furthermore, Gao, et al. [20,21] investigated the chemosensitivity of apigenin using BEL-7402/ADM cells, which are known for their resistance to DOX, a molecule belonging to the anthracycline family. Results showed that apigenin enhanced DOX sensitivity, induced apoptosis, and prevented HCC xenograft growth. Recently, treatment with apigenin was applied to ovarian cancer (OC) using ovarian cancer-sensitive cells (SKOV3) and drug-resistant cells (SKOV3/DDP) [22]. Results showed positive effects on the chemo-sensitivity of both cell types with apoptosis and reversal of drug resistance of these cancer cells through the down-regulation of the Mcl-1 gene.

#### 2.1.2. Flavanols

Quercetin is naturally distributed in many fruits, vegetables, leaves, seeds, and grains; capers, red onions, and kale contain appreciable quantities. Regarding quercetin, several research studies have evaluated the effect of this flavonoid against multidrug resistance by several mechanisms of action in various cancer cells. Research findings indicated that a co-treatment with quercetin combined with temozolomide (TMZ), an active anticancer drug, showed positive results such as inhibition of cell viability, induction of cell apoptosis, an increase of caspase-3 activity, elimination of drug insensitivity, and improvement of TMZ inhibition [23,27]. Some of these effects, such as the decrease in colony formation, inhibition of growth, and the stimulation of apoptosis by decreasing Bcl2 gene expression and increasing p53 and caspase-9 activity in esophageal (EC9706 and Eca109) have also been observed by combining quercetin with 5-FU [24] and breast (MCF-7) [34] cancer cells. 

The management of breast cancer attracted the attention of Li, et al. [25,28] who performed two experiments to evaluate the combination therapy of quercetin with DOX on MCF-7 cells. Results revealed that this treatment inhibits cell invasion and proliferation by suppressing the expression of HIF-1α and P-glycoprotein (P-gp), responsible for multidrug resistance [25]. In addition, results showed an increase in cellular apoptosis with down-regulation of p-Akt expression and up-regulation of phosphatase and tensin homolog (PTEN) expression [28]. Other effects of this combination, namely increasing cellular sensitivity to DOX and promoting DOX-induced cellular apoptosis via the mitochondrial/ROS pathway, respectively, have been noted in studies conducted by Chen, et al. [30] and by Shu, et al. [31] in the treatment of HCC (BEL-7402 cells) and prostate cancer (PC3 cells). Quercetin alone was able to down-regulate the expression of specific ABC transporters (ABCB1, ABCC1, and ABCC2) [30] with inhibition of the expression of the PI3K/AKT pathway [31].

On the other hand, the synergistic effect of quercetin was examined in vitro and in vivo with PTX, a molecule used in chemotherapy and synthesized by endophytic fungi [26]. Quercetin alone inhibited the proliferation of OC cells and increased their sensitivity to PTX [26]. Meanwhile, the combination of these two molecules showed an inhibition of the migration and proliferation of prostate cancer cells with an increase in apoptosis, and induction of G_2_/M cell cycle arrest, whereas the in vivo combination showed a synergistic effect in killing cancer cells [26]. Moreover, this flavonol positively affected GEM, a drug with significant cytotoxic activity, such as the improvement of cell death associated with increased caspase-3 and -9 activities in lung cancer cells. It caused considerable suppression of HSP70 chaperone protein expression compared to treatment with GEM alone [29]. This chemo-sensitivity has also been noted in pancreatic cancer cells [32]. In a recent study, Safi, et al. [35] evaluated the synergistic effect of quercetin (95 μM) with docetaxel (7 nM), an alkaloid with anticancer properties. Results revealed a decrease in STAT3, AKT, pERK1/2, and Bcl-2 proteins in MDA-MB-231 breast cancer cells. 

#### 2.1.3. Flavonols

Kaempferol is a flavonol found in numerous fruits and vegetables such as grapes, potatoes, squash, tomatoes, broccoli, onions, brussels sprouts, green beans, green tea, peaches, spinach, blackberries, lettuce, cucumber, apples, and raspberries. It has been studied for its chemo-sensitizing activity. Its association with quercetin has shown promising results, namely growth inhibition of adriamycin-resistant K562/A cells and myeloid leukemia K562 cells, increasing their sensitivity, and induction of apoptosis [36]. Additionally, this ubiquitous flavonoid chemo-sensitized 5-FU resistant colon cancer LS174-R cells and the combination of both substances provided a synergistic effect by inhibiting cell viability and inducing cell cycle arrest [37]. This was explained recently by Wu et al. [38] who attributed these results to the inhibition of PKM2-mediated glycolysis. The combinatorial effect of 5-FU with a flavonol was further evaluated (in vitro and in vivo) with myricetin against esophageal carcinoma [39]. Several favorable outcomes such as suppression of cell proliferation, increase in cell apoptosis and caspase-3 expression, and decrease in Bcl-2 and tumor xenograft growth (in vivo) were observed. In addition, kaempferol increased the PTX cytotoxicity with modulation of anti- and pro-apoptotic markers in OC cells [40].

As previously reported in breast cancer treatment with flavonoids, these secondary metabolites reverse cancer drug resistance and sensitize tumor cells to chemotherapy via several mechanisms. In this respect, Iriti et al. studied the chemo-sensitizing potential of rutin (3′,4′,5,7-Tetrahydroxy-3-[α-L-rhamnopyranosyl-(1–6)-β-D-glucopyranosyloxy]flavone) against two breast cancer cell lines (MB-MDA-231 and MCF-7 cells) [41]. At a dose of 20 μM, these researchers found that this flavonoid acts as a chemo-sensitizing agent by improving the anti-tumor effect of two chemotherapeutic agents (methotrexate and cyclophosphamide). Furthermore, rutin improved the in vivo efficacy of another anti-cancer drug (sorafenib) in a xenograft model of human HCC [42]. As seen with quercetin and berberine, another natural flavone called hispidulin enhanced cellular chemo-sensitivity by inhibiting the expression of the transcription factor HIF-1α via AMPK signaling in gallbladder cancer [43].

#### 2.1.4. Anthocyanidins

**Figure 4:**
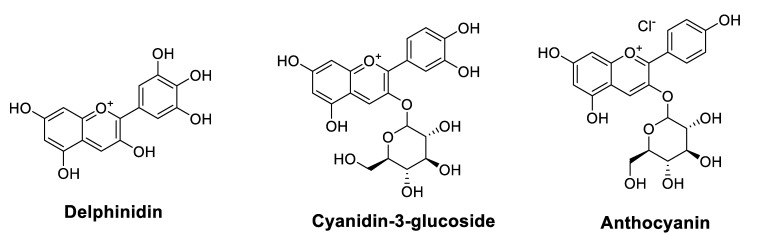
Chemical structures of anthocyanidins that improve chemosensitivity of anticancer drugs.

Anthocyanins (ACNs) are the primary color of many leaves (such as purple cabbage), fruits (such as grapes and blueberries), tubers (such as purple radishes and yams), and flowers (such as roses). In a broad sense, anthocyanidins (ACNs) present a subclass of flavonoids that have not been well investigated for their chemo-sensitizing and radio-sensitizing effects. Indeed, black raspberry ACNs improved the efficacy of two chemotherapeutic agents (5-FU and celecoxib); in vitro by inhibiting the proliferation of CRC cells and in vivo by decreasing the number of CRC tumors in animals [44]. Recently, specific molecules of this family, such as delphinidin [45] and cyanidin-3-glucoside (C3G) [46], have been studied. In radiation-exposed A549 human lung adenocarcinoma cells, delphinidin enhanced the radio-therapeutic effects (induction of autophagy and apoptosis) by activating the JNK/MAPK signaling pathway [45]. Similarly, C3G improved the sensitivity to DOX and its cytotoxicity by inhibiting the phosphorylation of Akt and increasing that of p38, mainly by reducing the expression of claudin-2 [46]. Table 2 lists anthocyanidins (Figure 4) that could improve the chemosensitivity of cancer drugs.

### 2.2. Non-Flavonoids

#### 2.2.1. Phenolic Acids 

**Figure 5:**
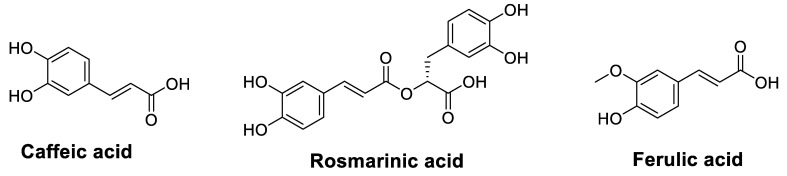
Chemical structures of phenolic acids that improve the chemosensitivity of anticancer drugs.

It has been demonstrated that phenolic acids have a chemo-sensitizing activity on several types of cancer cells to different chemotherapeutics (Table 3). Data presented in Table 3 indicate that ellagic acid was the most studied molecule. It is found in large quantities in pecans, chestnuts, raspberries, peaches, cranberries, strawberries, raw grapes, walnuts, and pomegranates. Indeed, its combination with 5-FU in treating colorectal carcinoma (CRC) gave significant effects such as inhibition of apoptotic cell death and cell proliferation. In contrast, treatment alone enhanced 5-FU chemo-sensitivity in CRC cells [47]. Indeed, ellagic acid alone potentiated CP cytotoxicity and prevented the development of CP resistance in epithelial OC cells [48]. Table 3 shows the phenolic acids (Figure 5) that improve the chemosensitivity of cancer drugs. 

Caffeic acid can be derived from a variety of beverages and is relatively present at high concentrations in lingonberry, thyme, sage, and spearmint as well as in spices such as Ceylon cinnamon and star anise. Caffeic acid is moderately available in sunflower seeds, applesauce, apricots, and prunes. Caffeic acid phenethyl ester (CAPE), a central component of propolis, has also been investigated for its chemo-sensitizing [51,52] and radio-sensitizing [53] effects against various types of cancer. The radio-sensitizing effect of this substance was evaluated in 2005 by Chen, et al. [49] against CT26 colorectal adenocarcinoma cells and in vivo on BALB/c mice implanted with these cells. These authors noted, in vitro, an improvement in the destruction of CT26 cells by ionizing radiation (IR) and, in vivo, an extension of animal survival and a marked inhibition of tumor growth compared to radiotherapy alone. The mechanism of action explaining this radio-sensitivity was elucidated very recently on prostate cancer cells (DU145 and PC3) by co-treatment using gamma radiation (GR) and CAPE [53]. Results showed that this combined treatment sensitizes the cells to radiotherapy by reducing the RAD50 and RAD51 proteins and the cell migration potential, mainly by inhibiting DNA damage repair. As for the chemo-sensitivity of this phenolic compound, Lin, et al. [50] did not observe any chemo-sensitizing effect of medulloblastoma Daoy cells on the chemotherapeutics studied (DOX or CP). However, in 2018, two similar studies proved otherwise by enhancing the sensitivity of gastric and lung cancer cells to DOX and CP by decreasing proteasome function [51,52].

In contrast, Muthusamy, et al. carried out two studies on the ability of ferulic acid (FA), a phenolic acid present in seeds and leaves of certain plants and found in exceptionally high amounts in popcorn and bamboo shoots, to reverse the resistance of multiresistant cells to anticancer drugs. In the first study, FA-enhanced cell cycle arrest was exerted by PTX and decreased resistance to this drug [54]. In the second study, FA increased VCR and DOX cytotoxicity and synergistically increased DOX-induced apoptotic signaling [55]. In addition, the authors showed that the synergy between FA and DOX reduced tumor xenograft size compared to the treatment with DOX alone. They associated these results with suppressing P-gp expression by inhibiting the PI3K/Akt/NF-κB signaling pathway.

Another phenolic acid constituent, called rosmarinic acid (RA), is found in culinary herbs such as *Ocimum tenuiflorum* (holy basil), *Origanum majorana* (marjoram), *Melissa officinalis* (lemon balm), *Ocimum basilicum* (basil), *Salvia officinalis* (sage), *Salvia rosmarinus* (rosemary), peppermint, and thyme. This natural compound showed remarkable potential as an anti-leukemic agent in acute promyelocytic leukemia cells by potentiating macrophage differentiation induced by all-trans retinoic acid [56]. Furthermore, Yu, et al. [57] evaluated the impact of RA on 5-FU chemo-resistance in the treatment of gastric carcinoma. In SGC7901 gastric carcinoma cells treated with 5-FU, the application of RA increased the chemo-sensitivity of these cells to 5-FU by reducing its IC_50_ values from 208.6 to 70.43 μg/mL and the expression levels of two miRNAs (miR-642a-3p and miR-6785-5p), with increased expression of FOXO4. 

#### 2.2.2. Tannins

**Figure 6:**
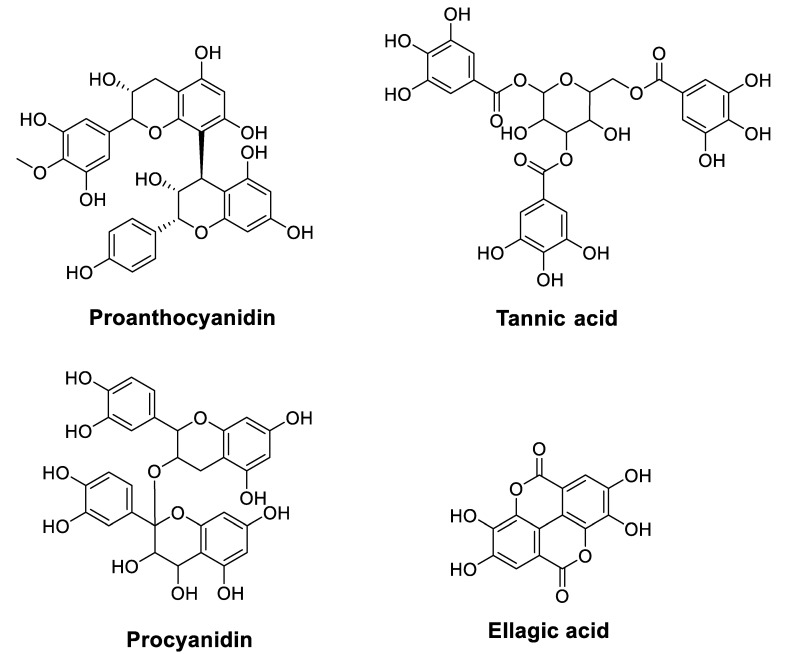
Chemical structures of tannins that improve chemosensitivity of anticancer drugs.

Although condensed tannins (also called proanthocyanidins (PCs)), found in plants, such as cranberry, blueberry, and grape seeds, are chemically polymers of flavanols, they have not been widely investigated as anticancer agents compared to flavonoids and phenolic acids. However, they have recently been studied to overcome the problems of cancer cell resistance to chemotherapy [58]. In this context, Zhang, et al. [58] showed that PCs inhibit the growth and characteristics of platinum-resistant OC cells by inducing G_1_ cell cycle arrest and targeting the Wnt/*β*-catenin signaling pathway. On the other hand, other researchers indicated that PCs sensitize chemoresistant CC cells (HCT116 and H716) to 5-FU and oxaliplatin (OXP) [59]. In contrast, combining all these substances reduced tumor growth in chemoresistant cells and chemoresistant tumor xenografts. The mechanism suggested to overcome this chemo-resistance involves suppressing the activity of adenosine triphosphate-binding cassette transporters. Furthermore, tannic acid (TA), another plant tannin used as an anticancer agent, has been studied for its synergistic effect with chemotherapeutic drugs (5-FU, GEM, and mitomycin C) against malignant cholangiocytes [60]. Results revealed that TA exhibits a crucial synergistic effect with 5-FU and mitomycin C in modulating drug efflux pathways. The exact synergy was observed by combining TA and CP on HepG2 liver cancer cells through mitochondria-mediated apoptosis [61]. This chemotherapeutic sensitivity to CP was corroborated by co-treatment with procyanidins in TU686 laryngeal cancer cells through the apoptosis and autophagy pathway [62]. Table 4 lists condensed tannins (Figure 6) that could improve the chemosensitivity of cancer drugs.

To improve the bioavailability and bioactivity of ellagic acid in vivo, Mady, et al. [63] formulated nanoparticles loaded with this acid from a biodegradable polymer [poly(ε-caprolactone)]. This encapsulation improved the oral bioavailability and the anti-tumor effect of ellagic acid. In a glioblastoma model, Cetin, et al. carried out two studies that showed an improvement in the anticancer efficacy of bevacizumab [64] and TMZ [65] by co-treatment with ellagic acid. This treatment reduced the expression of MGMT, affected caspase-3 and p53 proteins, and its combination with the chemotherapeutics reduced cell viability and the expression of MDR1.

Chemo-resistance of bladder cancer has been a serious problem in managing this type of cancer, particularly resistance to GEM. However, the underlying resistance mechanism has not been elucidated. The effect of ellagic acid or its combinatorial effect with GEM on GEM-sensitive bladder cancer cells and GEM-resistant cells was recently evaluated [66]. Results revealed that ellagic acid exerts numerous promising anticancer effects, particularly resensitization of GEM-resistant cells by inhibiting GEM transporters and the epithelial–mesenchymal transition (EMT), responsible for GEM resistance in other types of cancer. Suppression of EMT was also observed by catechol against pancreatic cancer cells, in addition to cellular chemo-sensitivity and radio-sensitivity to GEM via inhibition of the AMPK/Hippo signaling pathway [67].

## 3. Conclusions and Perspectives

At present, the use of foodstuffs is attracting attention in treating and preventing diseases, including cancer. This is due to the presence of bioactive compounds such as phenolic acids, among others, in our diet. These natural compounds are gaining popularity in cancer treatment due to their lower side effects, cost, and accessibility than conventional drugs. In this review, we have shown through published research that phenolic compounds are an excellent source of natural anticancer substances providing a range of preventive and therapeutic options against several types of cancer. These compounds could be used alone or in combination with other anticancer drugs. Certain phenolic compounds such as quercetin and gallic acid have well-known mechanisms of action. These molecules act specifically on the various checkpoints of cancerous cells. Therefore, exploring these mechanisms of action could further improve the therapeutic efficacy. However, further investigations that could involve human subjects and different pharmacokinetic parameters are required to ensure the safety of these compounds before they can be used as prescription drugs. In addition, the development of a standardized extract or dosage could also be followed in clinical trials. In summary, phenolic compounds present in our food can be useful in complementary medicine for the prevention and treatment of different types of cancers due to their natural origin, safety, and low cost compared to cancer drugs. 

## Figures and Tables

**Table 1:** Flavonoids improve the chemosensitivity of chemotherapeutic drugs in cancer.

Molecules	Origins	Experimental Approaches	Key Results	References
Apigenin	Synthetized	MDA435/LCC6 and P388 cells Cell proliferation assay ATPase assay	Enhanced the cytotoxicity of paclitaxel (PTX), doxorubicin (Dox), daunomycin (DM), vincristine (VCR), and vinblastine, resulting in a reduction of IC_50_ by 5–50 times	[11]
	Purchased	Parental human HCC cells (BEL-7402) and BEL-7402/ADM cells MTT assay Cell cycle analysis Real-time quantitative PCR Western blot analysis In vivo xenograft studies	Sensitized Dox-resistant BEL-7402 (BEL- 7402/ADM) cells to Dox Increased intracellular concentration of Dox Reduced Nrf2 expression APG + Dox (in vivo) inhibited tumor growth, reduced cell proliferation, and induced apoptosis more substantially when compared with Dox treatment alone	[12]
	Purchased	Human pancreatic cancer cell line BxPC-3 Human pancreatic ductal epithelium (HPDE) cells Western blot analysis MTS cell proliferation assay	APG (13 μM) + gemcitabine (Gem) (13 μM) inhibited cell proliferation APG (11–19 μM) + Gem (10 μM) inhibited growth by 59–73% Enhanced the anti-proliferative activity of chemotherapeutic drugs	[13]
	Purchased	Human pancreatic cancer cell lines AsPc-1, Panc-1, and MiaPaCa-2 MTT assay Cell apoptosis assay Western blot analysis In vitro IKK-β kinase activity assay Xenograft model	Reduced cell growth Induced cell apoptosis Down-regulated the TNF-α-induced NF-κB DNA binding activity Suppressed pancreatic cancer growth and IKK-β activation in nude mice xenograft	[14]
	Purchased	Laryngeal carcinoma Hep-2 cell line RT-PCR Cell counting Kit-8 (CCK-8) system Western blot analysis	Enhanced the cisplatin (CP)-induced suppression of Hep-2 cell growth in a concentration- and time-dependent manner Reduced the levels of GLUT-1 mRNA and GLUT-1 and p-Akt proteins in CP-treated Hep-2 cells in a concentration and time-dependent manner	[15]
	Purchased	Laryngeal hep-2 carcinoma cell line Nude mouse model of laryngeal carcinoma Western blot analysis	Improved xenograft radio-sensitivity Reduced the expression of PI3K mRNA, Akt, and GLUT-1 after X-ray radiation	[16]
	Purchased	Tumor xenografts in nude mice SK-Hep-1 and BEL-7402 cells MTT assay Annexin V/PI assay Western blotting analysis Cellular ROS detection	Enhanced the cytotoxicity of 5-FU in HCC cells APG + 5-fluorouracil (5-FU) (in vivo) inhibited HCC xenograft tumor growth APG + 5-FU increased the levels of reactive oxygen species (ROS) APG + 5-FU decreased the mitochondrial membrane potential (ΔΨm) APG + 5-FU decreased Bcl-2 expression	[17]
	Purchased	Human renal proximal tubular epithelial (HK-2) cells MTT assay Analysis of cell morphology and cell cycleCaspase-3 activity assay Western blot analysis ROS production assay	Inhibited the CP-induced apoptosis of HK-2 cells Induced cell cycle arrest Inhibited caspase-3 activity and PARP cleavage Reduced CP-induced phosphorylation and expression of p53 Promoted the CP-induced Akt phosphorylation	[18]
	Purchased	80 Swiss albino male mice ELISA Quantitative real-time RT-PCR Histopathological and immunohistochemical analysis	APG alone or combined with 5-FU Increased Beclin-1 levels, caspase-3 and -9, and JNK activities, decreased tumor volume, Mcl-1expression, and total antioxidant capacity, alleviated histopathological changes, and decreased Ki-67 proliferation index	[19]
	Not reported	BEL-7402 and BEL-7402/ADM cells TUNEL assay qRT-PCR Annexin V-FITC/PI apoptosis assay Western blot analysis	Reversed Dox sensitivity Induced the caspase-dependent apoptosis in BEL-7402/ADM cells Induced the miR-101 expression	[20]
	Not reported	Human hepatocellular carcinoma (HCC) and adjacent normal tissue specimens qRT-PCR MTT assay Western blot analysis In vivo xenograft studies	Enhanced Dox sensitivity Induced miR-520b expression Inhibited ATG7-dependent autophagy in BEL-7402/ADM cells Inhibited hepatocellular carcinoma xenograft growth	[21]
	Purchased	Ovarian cancer-sensitive cell line SKOV3 Ovarian cancer drug-resistant cell line SKOV3/DDP MTT assay PCR test Western blot test Apoptosis test	Enhanced the chemosensitivity of ovarian cancer-sensitive cells and drug-resistant cells Induced the apoptosis of ovarian cancer cells by down-regulating the Mcl-1 gene	[22]
Quercetin	Purchased	DB-1 melanoma and SK Mel 28 cell lines Western blot analysis Annexin V-FITC staining RNA isolation and RT-PCR Immunocytochemistry siRNA transfection	Induced a redistribution of ΔNp73 in the cytoplasm and nucleus Que + temozolomide (TMZ) abolished drug insensitivity and caused a more than additive induction of apoptosis	[23]
	Not reported	Human esophageal cancer cells (EC9706 and Eca109) MTT assay Annexin V-FITC/propidium iodide (PI)-stained fluorescence-activated cell sorting (FACS) Western blot analysis	Que + 5-FU inhibited growth and stimulated apoptosis in EC9706 and Eca109 esophageal cancer cells compared to Que	[24]
	Purchased	MCF-7 and MCF-7/Dox cells MTT assay Flow cytometry Matrigel invasion assay Western blot analysis	Increased intracellular concentration of Dox Improved Dox cytotoxicity Que + Dox inhibited cell proliferation and invasion and suppressed HIF-1α and P-gp expression	[25]
	Not reported	Human ovarian cancer cell lines, SKOV-3, EFO27, OVCAR-3, and A2780P Evaluation of quercetin toxicity SRB staining	Inhibited proliferation and increased sensitivity of ovarian cancer cells to CP and PTX	[26]
	Purchased	U251 and U87 human glioblastoma cells MTT assay Flow cytometry Western blot analysis	Que (30 μmol/L) + TMZ (100 μmol/L) inhibited cell viability and enhanced TMZ inhibition Que did not affect the caspase-3 activity and cell apoptosis, whereas combined with TMZ, it increased the caspase-3 activity and induced cell apoptosis.	[27]
	Purchased	MCF-7 cells and MCF-7/Dox cells MTT assay Flow cytometry	Que + Dox inhibited cell proliferation and invasion Que + Dox increased cell apoptosis Que + Dox up-regulated PTEN expression Que + Dox down-regulated p-Akt expression	[28]
	Purchased	Lung cancer cells (A549 and H460 cells) Western blot analysis	Reduced cell viability Suppressed HSP70 expression Improved Gem-induced cell death linked to increased caspase-3 and caspase-9 activities Que + Gem down-regulated HSP70 expression more significantly than treatment with Que or Gem alone	[29]
	Not reported	BEL-7402 and multidrug-resistant cell line BEL/5-FU MTT assay Flow cytometry Real-time PCR Western blot analysis	Increased intracellular accumulation of Dox Increased sensitivity of BEL/5-FU cells to chemotherapeutic drugs Down-regulated the expressions of ABCB1, ABCC1, and ABCC2 Inhibited the functions and expressions of ABCB1, ABCC1, and ABCC2 efflux pump	[30]
	Purchased	Human prostate cancer cell line PC3 MTT assay Western blot analysis Flow cytometry	Inhibited c-met expression and the downstream PI3K/AKT pathway Que + Dox promoted the Dox-induced cell apoptosis through the mitochondrial/ROS pathway	[31]
	Purchased	Human pancreatic cancer cell lines Transfection of small interfering RNA MTT assay Western blot analysis Cell cycle measurement	Attenuated RAGE expression to facilitate cell cycle arrest, autophagy, apoptosis, and GEM chemosensitivity in MIA Paca-2 GEMR cells	[32]
	Purchased	Human prostate cancer (PC-3) cell lines Nude male BALB/c mice MTT assay Intracellular ROS content assays RNA extraction and qRT-PCR Western blot analysis Immunohistochemistry	Que + PTX inhibited cell proliferation, increased apoptosis, arrested cell cycle at the G_2_/M phase, inhibited cell migration, induced ER stress, and increased ROS generation Que + PTX exerted the most beneficial therapeutic effects (in vivo) Increased the cancer cell-killing effects of PTX (in vivo)	[33]
	Not reported	MCF 7 cells MTT assay Flow cytometry qRT-PCR ELISA	Quer + 5-FU improved apoptosis by increasing the gene expression of Bax and p53 and caspase-9 activity and decreasing Bcl2 gene expression Quer + 5-FU decreased colony formation	[34]
	Purchased	MDA-MB-231 human breast cancer cell line MTT assay Flow cytometry qRT-PCR Western blot analysis	Decreased cell viability Que (95 μM) + docetaxel (7 nM) up-regulated p53, increased BAX levels, and decreased levels of BCL2, pERK1/2, AKT, and STAT3 proteins	[35]
Kaempferol	Purchased	Human myelogenous leukemia K562 cells and the adriamycin-resistant variant K562/A cells MTT assay Annexin V/PI analysis PCR array	Kae + Que inhibited the growth of both cells Kae + Que increased the sensitivity of both cells Kae + Que induced apoptosis Kae + Que influenced the expression of drug transporter genes	[36]
	Purchased	LS174 colon cancer cells MTT assay Colony formation assay Spheroid generation Sensitization assay Measurement of ROS Western blot analysis qRT-PCR	Chemo-sensitized 5-FU-resistant LS174-R cells Blocked the production of ROS and modulated the expression of JAK/STAT3, MAPK, PI3K/AKT, and NF-κBKae + 5-FU exerted a synergistic inhibitory effect on cell viability Kae + 5-FU enhanced apoptosis and induced cell cycle arrest in chemo-resistant and sensitive cells	[37]
	Purchased	Human colorectal cancer cell line HCT8 5-FU-resistant cell line HCT8-R CCK-8 assay qPCR assay Western blot analysis Clonogenic assay	Reversed the drug resistance of HCT8-R cells to 5-FU Reduced glucose uptake and lactic acid production in drug-resistant colorectal cancer cells Promoted the expression of microRNA-326 in colon cancer cells Reversed the resistance of colorectal cancer cells to 5-FU	[38]
Myricetin	Purchased	Esophageal carcinoma EC9706 cells Colony formation assays Flow cytometry Western blot analysis Nude mouse tumor xenograft model	MYR + 5-FU suppressed cell survival fraction and proliferation, and increased cell apoptosis MYR + 5-FU decreased survivin, cyclin D, and Bcl-2, and increased the expression level of caspase-3 and p53 MYR + 5-FU reduced the growth rate of tumor xenografts in mice	[39]
	Purchased	A2780 and OVCAR3 ovarian cancer cells MTT assay Apoptosis assay Boyden chamber assay Western blot analysis	Induced cytotoxicity, with an IC_50_ value of 25 μM Induced cell apoptosis, accompanied by the modulation of certain pro- and anti-apoptotic markers Increased paclitaxel cytotoxicity	[40]
Rutin	Purchased	Human breast cancer MDA- MB-231 cellsCalcein acetoxymethyl accumulation assayRhodamine-123 uptake assay Annexin V and 7-aminoactinomycin D Propidium iodide staining	Increased the anticancer activity of both chemotherapeutic agents Decreased the activity of adenosine triphosphate binding cassette transporters RTN (20 μM) enhanced cytotoxicity related to cyclophosphamide and methotrexate RTN (20 and 50 μM) arrested the cell cycle at the G_2_/M and G_0_/G_1_ phases, respectively, thus promoting cell apoptosis	[41]
	Purchased	Human HCC cell lines qRT-PCR Luciferase reporter assay Cell viability assay Flow cytometry In vivo tumor xenograft	Attenuated autophagy and BANCR expression in SO-resistant cells Decreased the number of autophagosomes in HepG2/SO and HCCLM3/SO cells Enhanced the efficacy of SO in a xenograft model of HCC in nude mice	[42]
Hispidulin	Not reported	Human gallbladder carcinoma cell line GBC-SD MTT assay Western blot analysis Flow cytometry Caspase-3 activity assay qRT-PCR In vivo xenograft experiments	Inhibited the growth of GBC cells Promoted apoptosis in GBC cells Induced cell arrest at the G_0_/G_1_ phase Exerted antitumor effect mediated through HIF-1α inhibition Repressed the transactivation activity and expression of HIF-1α Suppressed the HIF-1α expression via AMPK signaling	[43]

**Table 2:** Anthocyanidins that could enhance the chemosensitivity of cancer drugs.

Molecules	Origins	Experimental Approaches	Key Results	References
Delphinidin	Purchased	A549 cell line (human, lung, and carcinoma) MTT assay Immunofluorescence staining Western blot analysis qRT-PCR	Induced apoptosis in A549 cells Promoted apoptosis in the radiation-exposed A549 cells Induced autophagy in radiation-exposed A549 cellsActivated autophagic cell death and the JNK/MAPK signaling pathway in radiation-exposed A549 cells	[45]
Cyanidin-3-glucoside (C3G)	Purchased	Human lung adenocarcinoma A549 cells Immunoblotting RNA isolation and qRT-PCR Immunofluorescence measurement Luciferase reporter assay	Reduced protein level of CLDN2 in A549 cells Inhibited Akt phosphorylation Increased p38 phosphorylation Reduced CLDN2 expression at transcriptional and post-translational steps mediated by Akt inhibition and p38 activation, respectively Improved Dox accumulation and cytotoxicity in spheroid models Increased the percentages of apoptotic and necrotic cells induced by Dox	[46]
Anthocyanins (ACNs)	Black raspberry	Colon cancer cell lines, SW480 and Caco2MTT assay Colony formation assays Western blot analysis Establishment of colitis-induced colon cancer mice model	Improved the chemotherapy efficacy of 5-FU and celecoxib ACNs + (5-FU or celecoxib) inhibited CRC cell proliferation (in vitro) and decreased the number of tumors in AOM-induced CRC mice (in vivo)	[44]

**Table 3:** Phenolic acids that improve the chemosensitivity of cancer drugs.

Molecules	Origins	Experimental Approaches	Key Results	References
Caffeic acid phenethyl ester (CAPE)	Not reported	Mouse CT26 colorectal adenocarcinoma cells BALB/c mouse with CT26 cells implantation Colony formation assay RT-PCR Flow cytometry	Depleted intracellular GSH in CT26 cells, but not in bone marrow cells Enhanced cell killing by IR Increased glutathione peroxidase, decreased glutathione reductase in CT26 cells Reversed radiation-activated NF-κB Induced a significant inhibition of tumor growth and prolongation of survival compared to IR alone (in vivo)	[49]
	Purchased	Human medulloblastoma Daoy cell line and Human astroglia SVGp12 MTT and trypan blue exclusion assays ELISATUNEL assay Flow cytometry Western blot analysis	Inhibited Daoy cell growth in a time- and concentration-dependent manner Decreased G_2_/M fraction and increased S phase fractionDown-regulated expression of cyclin B1 protein Reduced the viability of irradiated Daoy cells No chemosensitizing effect on Dox or CP	[50]
	Purchased	Parental and the drug-resistant cells of stomach (MKN45) and colon (LoVo) cancers	Potentiated the apoptotic effects of Dox and CP against parental cells Reduced the production of Dox-induced ROS Reduced 26S proteasome-based proteolytic activities in parental MKN45 cells Up-regulated and significantly decreased chymotrypsin-like activity in Dox- or CP-resistant cells	[51]
	Not reported	Human lung adenocarcinoma A549 and RERF-LC-MS cell lines Immunoblotting RNA isolation and PCR Luciferase reporter assay Immunocytochemistry	Decreased claudin-2 protein level in a concentration-dependent manner Decreased (at 50 µM) mRNA level and promoter activity Decreased (at 50 µM) the level of p-NF−κB, and increased that of IκB Increased the expression and activity of protein phosphatase (PP) 1 and 2A Suppressed cell proliferation Enhanced Dox toxicity and accumulation in 3D spheroid cells	[52]
	Not reported	Prostate cancer (PCa) cells, DU145 and PC3 Evaluated the radiomodulatory potential of CAPE	CAPE + gamma radiation (GR) sensitized PCa cells to radiation in a concentration-dependent manner Improved the level of ionizing radiation (IR)-induced gamma H2AX foci and cell death by apoptosis CAPE + GR decreased the migration potential of PCa cells Sensitized PCa cells to radiation in vitro and induced apoptosis, increased Akt/mTOR phosphorylation and hampered cell migration CAPE + IR inhibited cell growth by decreasing RAD50 and RAD51 proteins	[53]
Ferulic acid (FA)	Purchased	Multidrug resistance (MDR) cell lines MTT assay Colony formation assay Fluorescence microscopic analysis Cell cycle analysis Tryptophan fluorescence quenching PCR array Western blot analysis	Inhibited P-glycoprotein transport function in drug-resistant KB ChR8-5 cell lines Down-regulated ABCB1 expression in a concentration-dependent manner Decreased paclitaxel resistance in KBChR8-5 and HEK293/ABCB1 cells Enhanced paclitaxel-mediated cell cycle arrest and up-regulated paclitaxel-induced apoptotic signaling in KB-resistant cells	[54]
	Purchased	Parental KB cells and P−gp overexpressing KB Ch^R^8-5 cell lines MTT assay γH2AX assay Western blot analysis Immunocytochemistry Animals and tumor xenograft experiments	Increased the cytotoxicity of Dox and VCR in the P-gp overexpressing KB Ch^R^8-5 cells Enhanced the formation of Dox-induced γH2AX foci and synergistically increased Dox-induced apoptotic signaling in drug-resistant cells FA + Dox reduced KB Ch^R^8−5 tumor xenograft size three-fold compared to the group treated with Dox alone Reversed MDR by suppressing P-gp expression via inhibition of PI3K/Akt/NF−κB signaling pathway	[55]
Rosmarinic acid (RA)	Purchased	Human acute promyelocytic leukemia NB4 cells Flow cytometry analysis Phagocytosis assay qRT-PCR	Potentiated ATRA-induced macrophage differentiation in APL cells	[56]
	Not reported	Human gastric carcinoma cell line SGC7901 Apoptosis assay CCK8 assay Apoptosis assay RNA isolation and microarray qRT-PCR Luciferase reporter assay Western blot analysis	Increased the chemosensitivity of SGC7901 cells to 5-FUReduced IC_50_ of 5-FU (70.43 ± 1.06 μg/mL) compared to untreated SGC7901/5-FU cells (208.6 ± 1.09 μg/mL) RA + 5-FU increased apoptosis rate Reduced the expression levels of two miRNAs (miR-642a−3p and miR−6785-5p) Reduced P-gp expression and increased Bax expression in SGC7901/5-FU and SGC7901/5-FU -Si cells	[57]

**Table 4:** Condensed tannins that could improve the chemosensitivity of cancer drugs.

Molecules	Origins	Experimental Approaches	Key Results	References
Proanthocyanidins	Chinese bayberry leaves	Platinum-resistant human ovarian cancer cell line OVCAR-3 Flow cytometry MTT assay Colony formation assay Western blot assay	Induced inhibitory effects on the growth and CSC characteristics of OVCAR−3 SP cells Reduced the expression of β-catenin, cyclin D1, and c-Myc and inhibited the self-renewal capacity of cells Induced G_1_ cell cycle arrest in OVCAR−3 SP cells	[58]
	Grape seed extract	Colorectal cancer cell lines, HCT116 and H716 Cell cycle and apoptosis analysis Cell viability and proliferation mRNA expression analysis Genome-wide RNA-sequencing analysisXenograft animal experiments	Sensitized acquired (HCT116-FOr cells) and innately chemoresistant (H716 cells) cancer cells to 5-FU and oxaliplatin (OXP) PCs + (5-FU and OXP) inhibited the growth of chemoresistant cells and decreased the expression of several key adenosine triphosphate-binding cassette (ABC) transporters Sensitized chemoresistant cells to 5-FU and OXPPCs + (5-FU and OXP) reduced chemoresistant xenograft tumor growth in mice	[59]
Tannic acid	Purchased	Malignant human cholangiocytes Calcein retention assays Western blot analysis RT-PCR	Decreased malignant cholangiocyte growth Exhibited a synergistic effect with mitomycin C and 5-FU but not with Gem Decreased calcein efflux and expression of PGP, MRP1, and MRP2 membrane efflux pumps	[60]
	Purchased	Liver cancer cell line HepG2 MTT assay Mitochondrial transmembrane potential qRT-PCR Western blot analysis	Inhibited HepG2 cell growth TA + CP induced mitochondria-mediated apoptosis in HepG2 cells and enhanced growth inhibitory effect compared to treatment alone	[61]
Procyanidins	Not reported	Laryngeal cancer cell line TU686 Flow cytometry Cell immunofluorescence staining Western blot analysis	Inhibited TU686 cells in a concentration-dependent manner for 24 h Induced apoptosis of TU686 cells Increased expression of LC3−Ⅱ and Caspase-3	[62]
Ellagic acid	Purchased	Colorectal carcinoma HT−29, Colo 320DM, SW480, and LoVo cells Trypan blue exclusion Annexin−V labeling Mitochondrial membrane potential (Δψm) Immunoblotting	EA + 5-FU inhibited cell proliferation of HT-29, Colo 320DM and SW480 cells EA + 5-FU increased apoptotic cell death of HT−29 and Colo 320DM cells EA potentiated 5-FU chemosensitivity in at least three colorectal cancer cell lines	[47]
	Purchased	Epithelial ovarian cancer cell line A2780MTT assay Immunoblot analysis Signal pathway analysis Cell cycle analysis	Enhanced CP cytotoxicity in A2780CisR cells Prevented the development of CP resistance	[48]
	Purchased	Caco-2 and HTC-116 cells MTT assay In vitro drug release Male New Zealand white rabbits	Induced higher cell viability than EA-NP treated HCT−116 cells Oral administration of EA-NPs caused a 3.6-fold increase in the area under the curve compared to that of EA (in vivo)	[63]
	Purchased	Rat C6 glioma cells Immunohistochemistry RT-PCR	Reduced MGMT expression Affected the apoptotic proteins of p53 and caspase-3 at the protein level, but not at the gene level EA + bevacizumab (BEV) reduced cell viability EA + BEV reduced MDR1 expression only at 72 h	[64]
	Purchased	Rat C6 glioma cells Immunocytochemistry RT-PCR	EA + TMZ reduced cell viability Down-regulated MGMT expression independent of the presence of TMZ EA + TMZ reduced MDR1 expression only over 48 h compared to TMZ alone Up-regulated caspase-3 at 48 h, but up-regulated p53 at 48 and 72 h EA + TMZ enhanced immunoreactivities of p53 and caspase-3 proteins, but not of the genes	[65]
	Purchased	Four human bladder cancer cell lines, TSGH−8301, TSGH-9202, T24, and J82 MTT assay Flow cytometry Cell migration and invasion assays Western blot analysis qRT−PCR Xenograft model	Induced high cytotoxicity of Gem in GEM−resistant cells EA + Gem increased apoptosis and reduced cell motility in GCB-resistant cells Resensitized bladder cancer cells to Gem by reducing the epithelial–mesenchymal transition Reduced EMT by inhibiting the TGFβ−SMAD2/3 upward signaling pathway Inhibited the growth of bladder cancer tumors and increased the in vivo inhibitory effects of Gen on tumors	[66]

## Abbreviations

**ACNs**	Anthocyanidins
**Akt**	Protein Kinase B
**APG**	Apigenin
**CAPE**	Caffeic Acid Phenethyl Ester
**CLL**	Chronic Lymphocytic Leukemia
**COX-2**	Cyclooxygenase-2
**CRC**	Colorectal Carcinoma
**DM**	Daunomycin
**DOX**	Doxorubicin
**ELISA**	Enzyme-linked immunosorbent assay
**EMT**	Epithelial-Mesenchymal Transition
**ERK**	Extracellular Signal-Regulated Kinase
**FA**	Ferulic acid
**GLUT-1**	Glucose Transporter-1
**Gem**	Gemcitabine
**GR**	Gamma Radiation
**HCC**	Hepatocellular Carcinoma
**HIF-1α**	Hypoxia-Inducible Factor-1α
**HO**	Heme Oxygenase
**HPD**	Hispidulin
**HPDE**	Human pancreatic ductal epithelium
**IL**	Interleukin
**IR**	Ionizing Radiation
**JNK**	C-Jun N-Terminal Kinase
**KAE**	Kaempferol
**MAPK**	Mitogen-Activated Protein Kinase
**MDR1**	Multidrug resistance protein 1
**MM**	Multiple Myeloma
**mTOR**	mammalian Target of Rapamycin
**MYR**	Myricetin
**NF-ĸB**	Nuclear Factor Kappa B
**Nrf2**	Nuclear factor erythroid-related factor 2
**NSCLC**	Non-Small Cell Lung Cancer
**OC**	Ovarian Cancer
**OXP**	Oxaliplatin
**P-gp**	P-glycoprotein
**PTEN**	Phosphatase and Tensin Homolog
**PTX**	Paclitaxel
**Que**	Quercetin
**ROS**	Reactive Oxygen Species
**RTN**	Rutin
**STAT3**	Signal Transducer and Activator of Transcription 3
**TA**	Tannic acid
**TNF-α**	Tumor Necrosis Factor-α
**TMZ**	Temozolomide
**VBL**	Vinblastine
**VCR**	Vincristine
**5-FU**	5-Fluorouracil
